# Visual assessment of commercial drivers in the South West Region of Cameroon

**DOI:** 10.1186/s12886-021-01909-3

**Published:** 2021-03-23

**Authors:** Brice Nguedia Vofo, Doris Ako Ayuk, Jacob Pe’er, Alain Chichom-Mefire, Nicholas Tendongfor, Eleanor Ngwe Nche

**Affiliations:** 1 Department of Ophthalmology, Hadassah-Hebrew University Medical Center, POB 12000, 91120 Jerusalem, Israel; 2 Department of Medicine, Faculty of Health Sciences, University of Buea, Buea, Cameroon

**Keywords:** Driving, Visual acuity, Road traffic crashes, Cameroon

## Abstract

**Background:**

Driving is a visually intensive task. In Cameroon, where the burden of road traffic deaths is high, visual assessment is not universally performed before the issuance of driver licenses. This study aims to assess the visual status of commercial drivers (CDs) in the southwestern region of Cameroon, and to find its relation to road traffic crashes (RTCs).

**Methods:**

This work was a cross-sectional community-based study on CDs in Limbe and Buea. Questionnaires were used to assess sociodemographic parameters, the incidence of RTCs, and self-reported visual status. Visual acuity (VA) was measured using a standard Snellen chart at 6 m. Statistical analysis was performed using descriptive methods: frequencies, the paired Student’s t-test, and the chi-square test.

**Results:**

Two hundred seven CDs were enrolled in this study, all of which were male, with a mean age of 41.8 ± 12.1 years. A total of 15.0% had undergone an eye exam prior to licensure, and 3.4% had undergone an eye exam within the past 10 years. The VA in the better-seeing eye of participants was less than 6/9 and 6/12 in 14.1 and 10.6% of CDs, respectively. Seventy-five percent of CDs with self-reported poor vision and 95% of CDs with VA < 0.5 had a history of RTCs compared to 55.8% of CDs with self-reported good vision and 55.7% of CDs with VA ≥ 0.5 (*p* < 0.05). Injuries from RTCs were more common in CDs with self-reported poor vision (81.1%) and in those with VA < 0.5 (90.5%) compared to CDs who self-reported good vision (55.8%) and those with VA ≥ 0.5 (55.7%) (*p* < 0.05).

**Conclusions:**

A large proportion of CDs did not undergo a visual assessment before the issuance or renewal of their driver licenses. A substantial number of CDs had poor vision in their better-seeing eye and suffered from RTCs and related injuries, which suggests that the visual status of CDs in Cameroon is related to the gruesome number of road traffic crashes and deaths in the country. Therefore, concerned authorities should consider making vision tests a necessary requirement for the obtention of driver licenses.

**Supplementary Information:**

The online version contains supplementary material available at 10.1186/s12886-021-01909-3.

## Background

Globally, mortality and life-long disability due to road traffic crashes (RTCs) are steadily increasing, with the greatest burden being in low- and middle-income countries (which account for 93% of all road traffic deaths) [1]. Road traffic injury is the eighth leading cause of death among all age groups, surpassing the number of deaths due to human immunodeficiency virus (HIV/AIDS), tuberculosis, and diarrheal diseases [2]. The worst rates of road traffic crashes (RTCs) are present in sub-Saharan Africa (26.6 deaths per 100,000 people). In Cameroon, there are 30.1 deaths per 100,000 people, as compared to 9.3 deaths per 100,000 people in Europe [1, 3]. Factors that contribute to these gruesome figures include impaired driving, speeding, driving under the influence of alcohol or drugs, and not using protective gear and seat belts, as well as unsafe roads and vehicles, inadequate traffic law enforcement and poor postcrash care [4].

Driving is a visually intensive task. Vision alone contributes to approximately 95% of driving-related information input [5]. It is expected that the visual function of a driver allows him/her to quickly read, understand and act on standard traffic control signs while moving at the maximum allowed speed under different lighting conditions [6]. Conditions that affect visual acuity, the breadth of visual fields and color vision will adversely affect driving [7]. Visual acuity (VA) is an important aspect of visual function, playing an important role in driver safety and performance [8]. The widely accepted consensus is that nonprofessional drivers should have a visual acuity of at least 0.5 (6/12) in their better-seeing eye, with other specific requirements like visual fields and contrast sensitivity differing across countries and types of driver licenses [9]. There is no global consensus on the basic visual requirements for commercial drivers, but it is widely accepted that commercial drivers should have more stringent requirements compared to noncommercial drivers.

In developing countries, roads are the major means of transportation, and commercial driving is responsible for the burden of road traffic crashes [10, 11]. Factors inherent to drivers, together with environmental conditions, contribute to these crashes. Alcohol consumption and the use of CNS stimulants like kolanuts, marijuana, cigarettes and illicit drugs by CDs are significantly associated with the incidence of RTCs [12, 13]. The aggravating behavioral factors self-reported by commercial drivers include fatigued driving due to work pressures, speeding, the inadequate maintenance of vehicles, the lack of seat belt use, and distracted driving [14, 15]. In Ghana, for instance, 62% of CDs reported the use of a phone while driving [16]. Traffic sign deficits, bad roads and poor lighting also lead to high crash risk among CDs [17, 18]. Driving with poor vision is a potential hazard to oneself and other road users. An estimated 29.5% of Spanish drivers present with a visual issue that affects their driving [19], and several studies have shown high rates of RTCs in drivers with poor vision [20–24]. Verma et al. showed a significant relationship between RTCs and visual field defects in CDs [20], while Oladehinde et al. reported that 85.7% of CDs with visual acuity < 0.3 (6/18) had been involved in an RTC, compared to 24.5% of CDs with visual acuity > 0.5 (6/12) [25]. In developing countries, uncorrected refractive errors are the most common cause of visual impairment among CDs (7.6–31.3%) [22, 26], but these drivers either do not possess [12, 26] or do not use the appropriate refractive correction [21]. Other common visually impairing eye conditions found in CDs include cataracts, glaucoma, pterygia and color vision deficiencies [21, 24, 26–28].

It is therefore imperative to set minimal vision requirements before the issuance or renewal of a driver’s license [29]. Currently, minimum visual requirements are neither clearly established nor reinforced for the acquisition of driver licenses in Cameroon. A medical certificate validating an individual’s fitness to drive is, however, a requirement, but physicians are not obliged to perform a visual acuity exam [30]. There are also no minimum visual requirements for commercial drivers (CDs). These lapses prompted this study, aimed at assessing the visual status of commercial interurban and intraurban drivers in the southwestern region of Cameroon and its relation to road traffic crashes.

## Methods

This was a cross-sectional descriptive study on the visual acuity assessment of CDs in the Limbe and Buea subdivisions of the Fako Division of the southwestern region of Cameroon. Limbe is a coastal city with many touristic attractions, and Buea is a mountainside city that houses one of the largest universities in Cameroon. There is significant traffic within and between these two cities, which are located approximately 40 km apart and both situated approximately 75 km from Douala, the largest city in Cameroon. Our target population was commercial intracity and intercity drivers who were ≥ 18 years old and consented to participate in the study. We excluded any individual who was sick or did not consent to participating in the study. The study was carried out from January to April 2020.

The minimum sample size was estimated using the Lorenz formula as follows:

$$ N=\left[p\Big(1-p{(z)}^2\right]/{d}^2 $$

where.

N = minimum sample size required for this study;

P = expected prevalence in the population. We obtained our P from a study carried out in the north-central state of Nigeria, which showed a prevalence of 9.1% (visual acuity < 6/18) in the better eye without correction (10);

d = precision = 0.05; and

z = coefficient of significance = 1.96.

Therefore, the minimum sample size *N* = 128 drivers.

For convenience, by using consecutive nonprobability sampling, we approached and recruited commercial motor drivers that met the inclusion criteria. They were given a standard questionnaire (Supplement 1) to complete, and their visual acuity was assessed using a standard Snellen chart mounted 6 m away from participants in a well-lit private setting. Each eye was assessed separately by occluding the other, and participants could put on any form of refractive correction they used while driving, if available.

The questionnaire had five sections covering participant identification, sociodemographic data, driver license acquisition, driving safety and visual acuity assessment. Approximately 8 minutes was required to complete each questionnaire. Serial numbers were used to identify participants, and the data obtained included age, educational level, marital status, possession of a valid driver’s license, driving tests done, eye tests and their frequency, involvement in road traffic crashes and their frequency over the previous 10 years, cause of the crash, and self-reported visual status. The questions were mainly close-ended multiple choice and checkbox questions, using a 5-point Likert scale wherever appropriate. The data were then collected on printed paper forms, transferred to Microsoft Excel sheets, coded, and stored in a password-protected computer.

Ethical clearance was obtained from the Institutional Review Board of the Faculty of Health Sciences, University of Buea, and the study was conducted according to the tenets of the Declaration of Helsinki. Statistical analysis was performed using SPSS version 25.0 (IBM Corporation, Somers, NY). The frequency counts and percentages that were generated were appropriate. A paired Student’s t-test was used to compare means, and a chi-square test was used to compare proportions. Statistical significance was set at a *p*-value of less than 0.05.

## Results

Two hundred seven (207) commercial drivers were interviewed and examined, 104 (50.2%) of which were taxi drivers, 56 (27.1%) of which were bus drivers, and 47 (22.7%) of which were truck drivers. They were all male, and their mean age was 41.8 ± 12.1 years (range 22–72 years). Most drivers (56.5%) had attained at least a secondary level of education (no education, 5.8%; primary 37.7%; secondary, 30.4%; tertiary, 16.9%; and vocational training, 9.2%), and 66.2% were married (Table 1).

**Table 1:** General Characteristics of Commercial Drivers

Characteristic		Frequency (n)	Percentage (%)
Age group (years)	20–30	35	16.9
	31–40	79	38.2
	41–50	43	20.8
	51– 60	36	17.4
	> 60	14	6.8
Education	None	12	5.8
	Primary	77	37.2
	Secondary	64	30.9
	Tertiary	35	16.9
	Vocational	19	9.2
Marital status	Divorced	3	1.5
	Married	137	66.2
	Single	61	29.5
	Widowed	6	2.9
Type of vehicle	Bus	56	27.1
	Taxi	104	50.2
	Truck	47	22.7

Even though 89.4% of the drivers possessed driver licenses, only 62.8% had ever taken a driving test. A total of 73.4% had renewed their license on its expiration at some point. A total of 15.0% had undergone an eye exam with visual acuity testing prior to licensure, and 3.4% reported having undergone an eye examination within the 10 years prior to the study.

VA in the better-seeing eye was assessed to be less than 6/9 and 6/12 in 14.1 and 10.6% of participants, respectively. None of these CDs wore spectacles or had other forms of refractive correction. Table 2 shows the distribution of their visual acuity in their better- and worse-seeing eyes.

**Table 2:** Visual Acuity Distribution in the Better and Fellow Eyes of Commercial Drivers

Visual Acuity	Better Eye (%)	Worse Eye (%)
**≥ 6/9**	177 (85.9)	145 (70.9)
**< 6/9 ≥ 6/12**	8 (3.9)	33 (15.9)
**< 6/12 ≥ 6/18**	15 (7.2)	16 (7.7)
**< 6/18 ≥ 6/24**	6 (2.9)	0 (0)
**< 6/24 ≥ 6/36**	1 (0.5)	12 (5.8)
**Total**	**207 (100)**	**207 (100)**

Approximately one-quarter (24.6%) of all the CDs self-reported poor vision. The characteristics and rates of accidents and injuries among them were compared to those of other drivers (Table 3).

**Table 3:** Comparison of CDs with Self-Reported Poor Vision and those with Self-Reported Good Vision

Characteristic	Self-reported poor vision	Self-reported good vision	***p***-value
**Number**	51 (24.6%)	156 (75.4%)	
**Mean age**	53.53 ± 12.29	37.97 ± 9.21	0.000
**History of RTCs**	37 (72.5%)	87 (55.8%)	0.048
**Mean number of RTCs over the past 10 years**	1.75 ± 1.64	1.03 ± 1.40	0.003
**Number of injuries**	7 (18.9%)	49 (56.3%)	0.000
**Mild injury**	28 (75.7%)	34 (39.1%)	0.000
**Severe injury**	2 (5.4%)	4 (4.6%)	1.000
**Mean LogMar VA**	0.2603 ± 0.24	0.0283 ± 0.09	0.000
**Proportion that took a driving test**	42 (82.4%)	88 (56.4%)	0.001
**Mean number of years driving**	29.10 ± 13.02	14.20 ± 8.74	0.000
**Proportion with a level of education above primary**	42 (82.4%)	75 (48.1%)	0.000

*VA* Visual acuity

CDs with self-reported poor vision had lower mean vision in their better-seeing eye (LogMar 0.26, ≈ Snellen 0.5) than did CDs who self-reported good vision (LogMar 0.028 ≈ Snellen 1.0). Those with poor vision were older (mean age 53.53 ± 12.29 years, *p* = 0.00) and had more driving experience (mean number of years driving 29.10 ± 13.02). CDs with self-reported poor vision were involved in a significantly higher number of RTCs (72.5%) than were CDs with self-reported good vision (55.8%, *p* = 0.048), and they also had a higher average number of accidents over the previous 10 years (1.75 ± 1.64) compared to CDs with self-reported good vision (1.03 ± 1.40, *p* = 0.003). CDs with VA < 0.5 were older (mean age of 57.50 ± 12.633 years compared to a mean age of 39.95 ± 10.56 in CDs with visual acuity > 0.5) and likeCDs with self-reported poor vision had worse parameter values when compared to CDs with VA ≥ 0.5. Ninety-five percent (95%) of CDs with VA < 0.5 had a history of an RTC over the past 10 years compared to CDs with VA > 0.5 (*p* = 0.000) and a higher average number of RTCs (2.91 ± 1.72) compared to CDs with VA > 0.5 (1.01 ± 1.33, *p* = 0.00). A total of 81.1% of CDs with VA < 0.5 (81.1%) sustained an injury in an RTC compared to 55.7% of CDs with VA > 0.5 (*p* < 0.05) (Table 4).

**Table 4:** Comparison of CDs with VA < 0.5 and CDs with VA ≥ 0.5 in their Better-Seeing Eye

Characteristic	VA < 0.5	VA ≥ 0.5	***p***-value
**Number**	22 (10.6%)	185 (89.4%)	
**Mean age**	57.50 ± 12.633	39.95 ± 10.56	0.000
**History of RTCs**	21 (95.5%)	103 (55.7%)	0.000
**Mean number of RTCs over the past 10 years**	2.91 ± 1.72	1.01 ± 1.33	0.000
**No injury**	2 (9.5%)	54 (52.4%)	0.000
**Mild injury**	14 (66.7%)	48 (46.6%)	0.044
**Severe injury**	5 (23.8%)	1 (1.0%)	0.000
**Proportion that reports having poor vision**	19 (86.4%)	32 (17.3%)	0.000
**Proportion that took a driving test**	13 (59.1%)	117 (63.2%)	0.816
**Proportion that possesses valid driver licenses**	20 (90.9%)	165 (89.9%)	1.000
**Mean number of years driving**	37.00 ± 9.23	15.71 ± 10.02	0.000
**Proportion with a level of education above primary**	16 (72.7%)	101 (54.6%)	0.117

*VA* Visual acuity, *CD* Commercial driver.

**Fig. 1:**
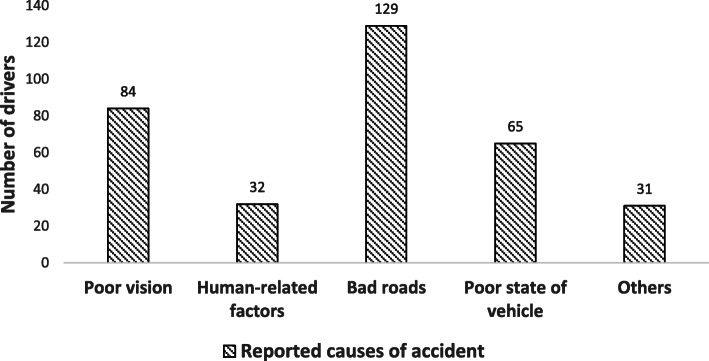
Self-Reported Causes of Accidents Among Commercial Drivers

According to CDs, the most common causes of RTCs were bad roads, poor vision, poor state of vehicles and human-related factors like insobriety (Fig. 1).

## Discussion

We assessed the visual acuity (VA) of commercial drivers (CDs) in a setting where there was no rule enforcing the visual assessment of drivers before the issuance or renewal of driver licenses. In such settings, bad driving conditions and poor road infrastructure may require even better visual abilities than in developed countries to ensure safe driving. In this study, which included 207 drivers, all drivers were male, with a greater proportion (38.2%) being in the 31- to 40-year age group (mean age: 41.8 ± 12.1 years). Young males generally make up the bulk of commercial drivers in Africa [31–33]. Our findings are similar to those of Verma et al. in an Indian population, who reported that all 288 bus and freight vehicle drivers in their study were male and that more than 65% of them were younger than 45 years [20]. In the developing world, high-risk and physically demanding occupations like commercial driving are generally left to relatively younger and stronger men, who can drive long hours, manage vehicle maintenance and handle passengers’ luggage. We found that a small proportion of respondents (5.8%) had no formal education, meaning that they might not have been able to read road signs. Most (57%) of the drivers had attained a secondary level of education, with up to 16.9% having attained university education. Our findings are similar to those reported by Okafor et al. in a highly literate community in Nigeria [33] and in contrast to those of other studies conducted in Africa [32–34]. This study area abodes the first Anglo-Saxon University of Cameroon, which can explain the higher literacy level of commercial drivers in the region, who may likely be university dropouts or graduates who did not find white-collar jobs.

Although over 89.4% of the drivers possessed valid driver licenses, only 62.8% had taken a driving test, and 15% percent had undergone an eye examination. This means that a significant proportion of commercial drivers did not take a driving test (37.2%) and that 85% did not undergo a visual assessment examination. Comparable results were seen in a Nigerian study where Chidi-Egboka et al. reported that although 97.7% of drivers had formal driver licenses, only 83.5% had undergone a formal driving test [31]. This could be due to the constant policing of the forces of law and order on the roads, making it difficult to drive without a valid driver’s license and highlighting the ease with which these drivers can obtain a driver’s license without fulfilling official state requirements. However, in both this study and that of Chidi-Egboka et al. [31], less than one-third of those who had taken a formal driving test had their eyes examined. CDs who had taken a formal driving test could be considered those who were willing to comply with rules and regulations, but if those rules did not mandate eye examinations as an integral part of assessing their general fitness to drive, then it is expected that people with poor vision would drive while having valid licenses.

VA in the better-seeing eye in our study was assessed to be less than 6/9 and 6/12 in 14.1 and 10.6% of the drivers, respectively. Our findings are comparable to those found in other neighboring developing countries, where there are clearly regulations stipulating a minimum required VA of 6/9 in the better-seeing eye to drive. In Nigeria, Dairo et al. reported that 15.3 and 9.1% of intracity commercial drivers in Ibadan had VA of less than 6/9 and 6/12, respectively, in their better-seeing eye [35], while in Ghana, Ovenseri-Ogomo et al. reported that 12.1% of commercial vehicle drivers in the Cape Coast municipality had VA of less than 6/9 in their better-seeing eye [32]. Despite the existence of regulations in these countries, Nwosu reported that in Nigeria, driver licenses were mostly issued without visual assessment [36], implying the existence of regulations but without adequate measures to enforce them.

According to the CDs in this study, poor vision was the second most common cause of RTCs. Approximately one-quarter (24.6%) of the drivers reported that their vision was poor and were found to have significantly lower VAs in their better-seeing eyes. This group also had a higher proportion of individuals who had completed at least the secondary level of education. This association can be explained by the fact that higher levels of education result in a higher sense of self-awareness and ease of reporting possible deficits. It is also encouraging because this group of drivers can easily be educated to adopt better eye-health practices and behaviors. Interestingly, when we compared the characteristics of those who had a VA < 0.5 with those with a VA ≥ 0.5 in their better-seeing eye, it was similar to the comparison between CDs with self-reported poor vision and those with self-reported good vision. CDs who reported poorer vision and those with VA < 0.5 were older, had been driving for longer periods, had a higher number of reported RTCs over the last 10 years, and sustained more injuries. Seventy-five percent of CDs with self-reported poor vision and 95% of CDs with VA < 0.5 had a history of RTCs compared to 55.8% of CDs with self-reported good vision and 55.7% of CDs with VA ≥ 0.5 (*p* < 0.05).

The link between lower visual acuity in drivers and a higher occurrence of RTCs has been described [22, 25, 37]. In India, among 387 drivers, 81.9% of road traffic crashes were seen in bus and freight drivers compared to private vehicle drivers. In this same study, commercial drivers with a visual acuity less than < 0.5 had a higher percentage of crash involvement (> 87%) [20]. Given that vision is likely to decrease with age [38], it may be understood that older drivers have poorer vision compared to younger drivers. This could be linked to associated age-related sight-threatening conditions like cataracts, advanced glaucoma, uncorrected refractive errors, macular diseases, and a lower propensity for visual healthcare. We did not perform eye exams in this study. None of the drivers had a refractive correction, and uncorrected refractive errors (often more common in the older population) have been identified as the most important cause of low vision in similar settings [39]. Ovenseri-Ogomo et al. also reported in their study that only 4.9% of the CDs in the Cape Coast municipality in Ghana wore spectacles (70% of whom bought spectacles from roadside stands), and refractive errors were identified as the most common cause of poor vision among these drivers [32]. In this study, 55% of CDs with VA ≥ 0.5 were still involved in an RTC, meaning vision-related issues alone contributed to 40% of RTCs. However, these figures are still high and similar to reports by Verma et al. in India [20], suggesting that visual acuity alone, though it greatly contributes to RTCs, is not the only factor involved. Drivers reported that bad roads were the main cause of RTCs, followed by poor vision. Injuries from RTCs were more common in CDs with self-reported poor vision (81.1%) and in those with VA < 0.5 (90.5%) compared to CDs who self-reported good vision (55.8%) and those with VA ≥ 0.5 (55.7%) (*p* < 0.05). CDs with poor vision are therefore more likely to be involved in road traffic crashes and their associated injuries than are CDs with good vision.

In studies where a visual field assessment was performed using standard perimetry, a strong association was found between visual field defects and the occurrence of road traffic crashes [40]. In this study, visual field assessment was not performed; however, a high proportion of drivers (24.6%) reported that they had poor vision, although a lower proportion was identified to have poor vision on examination (14.1%). This finding suggests that VA measures alone may not be sufficient for screening drivers. Visual field assessments should be considered in vision screening, especially for professional drivers. Vision screening and frequent eye tests among CDs, together with policies that allow only CDs with good vision to drive, will not only reduce the number of CDs with poor vision on the roads but will go a long way toward reducing the number of road traffic crashes and associated morbidity and mortality.

Our study was limited in that we used visual acuity as the only measure of visual health. The inclusion of visual fields, color vision, contrast sensitivity, and refractive tests would have provided more information on the visual status of participants. Due to technical difficulties, we could not perform these procedures. Future studies should consider full eye examinations and the associated ocular conditions. We also relied on the reports of drivers to ascertain their involvement in road traffic crashes and associated injuries, but such information could be affected by recall bias.

## Conclusions

This study brings to light the visual state of commercial drivers in Cameroon. To the best of our knowledge, this is the first study to assess the visual function of drivers in Cameroon and its relation to RTCs. A large proportion of commercial drivers possess driver licenses despite their inadequate visual abilities. Poor vision is a contributing factor to impaired driving and will adversely affect the incidence of road traffic crashes and related mortality and morbidity. These findings may be allegorical of settings with no established rules regarding visual assessments and driving. CDs are greatly responsible for the safety of many road users, including pedestrians and their passengers. Their poor visual status and associated higher involvement in RTCs will not only affect them but also put the life of every road user at risk. To attain the sustainable development goals for road safety by 2030 (to provide access to safe, affordable, accessible, and sustainable transport systems for all), the introduction and implementation of a compulsory minimum requirement for visual function before the obtention of a driver’s license in Cameroon and similar settings are essential. Drivers in general, especially commercial drivers, must undergo frequent eye exams to diagnose and treat vision-impairing ocular diseases, and drivers who cannot meet the minimum driving requirements should have their licenses revoked.

## Supplementary Information

**Additional file 1.** Study Questionnaire.

## Data Availability

The datasets used and/or analyzed during the current study are available from the corresponding author on reasonable request.

## Abbreviations

AIDS: Acquired immunodeficiency syndrome
CDs: Commercial drivers
HIV: Human immunodeficiency virus
RTC: Road traffic crash
VA: Visual acuity
