# Supplementary material for: Visual assessment of commercial drivers in the South West Region of Cameroon
Source: BMC Ophthalmol. 2021 Mar 23;21:148. doi: 10.1186/s12886-021-01909-3 (PMC7986018; doi:10.1186/s12886-021-01909-3)
Supplement: Supplementary file 1 — Additional file 1. Study Questionnaire. [file 12886_2021_1909_MOESM1_ESM.docx]

**STUDY QUESTIONNAIRE**

**VISUAL ASSESSMENT OF COMMERCIAL DRIVERS IN THE SOUTH WEST REGION OF CAMEROON**

**Brice Nguedia Vofo^1^, Doris Ako Ayuk^2^, Jacob Pe’er^1^ Alain Chichom-Mefire ^2^, Nicholas Tendongfor^2^, Eleanor Ngwe Nche^1^.**

**^1^** Department of Ophthalmology, Hadassah-Hebrew University Medical Center, Jerusalem, Israel.

**^2^** Department of Medicine, Faculty of Health Sciences, University of Buea, Buea, Cameroon.

**Corresponding author:** Eleanor N. Nche. Department of Ophthalmology, Hadassah-Hebrew University Medical Center, POB 12000, Jerusalem 91120, Israel. Telephone: +972-2-677-6365. Mobile: +972-50-404-8879. Fax: 02- 6778297. Email: [eleanor.nche@gmail.com](mailto:eleanor.nche@gmail.com).

**RUNNING TITLE:**

**VISUAL STATUS OF COMMERCIAL DRIVERS IN CAMEROON**

**SECTION A: IDENTIFICATION**

| A1 | **S**erial number |  |  |
| --- | --- | --- | --- |
| **A2** | Date of interview |  |  |
| A3 | Telephone number/email |  |  |

**SECTION B: SOCIO-DEMOGRAPHIC DATA**

| B1 | Age | 18-29 | 30-39 | 40-49 | 50-59 | 60+ |
| --- | --- | --- | --- | --- | --- | --- |
| B2 | Educational level | None | Primary | Secondary | Tertiary | Vocational training |
| B3 | Marital status | Single | Married |  |  |  |
| B4 | Religion | Christianity | Islam | Others |  |  |
| B5 | Tribe |  |  |  |  |  |

**SECTION C: DRIVING LICENSE ACQUISITION**

| C1 | Type of vehicle | | | | | | |
| --- | --- | --- | --- | --- | --- | --- | --- |
| C2 | | Age of first issue of license | | | |  |  |
| C3 | | Do you currently possess a valid driving license? | | | | Yes | No |
| C4 | | Was a driving test conducted prior to obtaining your license? | | | | Yes | No |
| C5 | | Was any eye examination done by a medical doctor prior to obtaining your license? | | | | Yes | No |
| C6 | | Have you ever renewed your driving license upon expiration? | | | | Yes | No |
| C7 | | If yes on C6, how many times. If no skip to section D. | | | |  |  |
| C8 | | was any eye examination done upon renewal | | | | Yes | No |
| C9 | | How frequent do you get your eyes checked by a doctor? | none | At least once a year | At  least once every three years | At  least once every ten years | At  least once every twenty years |

**SECTION D: DRIVING SAFETY**

| D1 | Have you been involved in a road traffic crash in the past? | Yes | No |  |  |  |
| --- | --- | --- | --- | --- | --- | --- |
| D2 | Number of road traffic crash in the last ten years? | None | Once | Twice | Thrice | specify |
| D3 | Seriousness of the crash | No injury | mild | Severe | Fatal |  |
| D4 | Do you think you have poor vision? | Yes | no |  |  |  |
| D5 | Cause of the crash | Visual | human | Road | Vehicle | Others |

**SECTION E: VISUAL ACUITY ASSESSMENT**

| E1 | Visual acuity in the better eye |  |  |
| --- | --- | --- | --- |
| E2 | Visual acuity in the second eye |  |  |
|  |  |  |  |
